# The bisphenol S contamination level observed in human follicular fluid affects the development of porcine oocytes

**DOI:** 10.3389/fcell.2023.1145182

**Published:** 2023-04-06

**Authors:** Tereza Žalmanová, Kristýna Hošková, Šárka Prokešová, Jan Nevoral, Michal Ješeta, Michal Benc, Young-Joo Yi, Jiří Moravec, Beáta Močáryová, Stanislava Martínková, Josef Fontana, Moustafa Elkalaf, Jan Trnka, Jana Žáková, Jaroslav Petr

**Affiliations:** ^1^ Department of Biology of Reproduction, Institute of Animal Science, Prague, Czechia; ^2^ Biomedical Center, Faculty of Medicine in Pilsen, Charles University, Pilsen, Czechia; ^3^ Department of Histology and Embryology, Faculty of Medicine in Pilsen, Charles University, Pilsen, Czechia; ^4^ Department of Obstetrics and Gynecology, Faculty of Medicine, Masaryk University and University Hospital Brno, Brno, Czechia; ^5^ Faculty of Natural Sciences and Informatics, Constantine the Philosopher University of Nitra, Nitra, Slovakia; ^6^ Department of Agricultural Education, College of Education, Sunchon National University, Suncheon, Republic of Korea; ^7^ Institute of Biology and Medical Genetics, First Faculty of Medicine, Charles University, Prague, Czechia; ^8^ Department of Biochemistry, Cell and Molecular Biology, Third Faculty of Medicine, Charles University, Prague, Czechia; ^9^ Centre for Research on Diabetes, Metabolism and Nutrition, Third Faculty of Medicine, Charles University, Prague, Czechia; ^10^ Department of Pathophysiology, Third Faculty of Medicine, Charles University, Prague, Czechia

**Keywords:** bisphenol S (BPS), oocyte, aneuploidy, meiosis, follicular fluid (FF), endocrine disruption, embryonic development

## Abstract

Bisphenol S (BPS), the main replacement for bisphenol A (BPA), is thought to be toxic, but limited information is available on the effects of Bisphenol S on ovarian follicles. In our study, we demonstrated the presence of Bisphenol S in the follicular fluid of women at a concentration of 22.4 nM. The effect of such concentrations of Bisphenol S on oocyte maturation and subsequent embryo development is still unknown. Therefore, we focused on the effect of Bisphenol S on *in vitro* oocyte maturation, fertilization, and embryo development. As a model, we used porcine oocytes, which show many physiological similarities to human oocytes. Oocytes were exposed to Bisphenol S concentrations similar to those detected in female patients in the ART clinic. We found a decreased ability of oocytes to successfully complete meiotic maturation. Mature oocytes showed an increased frequency of meiotic spindle abnormalities and chromosome misalignment. Alarming associations of oocyte Bisphenol S exposure with the occurrence of aneuploidy and changes in the distribution of mitochondria and mitochondrial proteins were demonstrated for the first time. However, the number and quality of blastocysts derived from oocytes that successfully completed meiotic maturation under the influence of Bisphenol S was not affected.

## 1 Introduction

Endocrine disruptors (EDs) are a group of harmful substances that enter the environment mainly through anthropogenic activity (summarized in Goralczyk, 2021) and are able to disrupt the hormonal control of an organism even at very low concentrations (Colborn et al., 1993). One of the most widespread EDs is bisphenol A (BPA), which has been detected in water, air and soil (summarized in Vasiljevic and Harner, 2021). Its negative effect on the health and reproduction of humans and animals has been clearly demonstrated EFSA (European Food Safety Authority), 2011; FDA (Food and Drug Administration), 2012; Vandenberg and Pelch, 2021.

For this reason, BPA production faces many regulations and is being reduced. Bisphenol S (BPS), an unvetted alternative to BPA, is used in daily activities more often, even though recent studies suggest that it has more serious health consequences than BPA (Mao et al., 2020; Darghouthi et al., 2022). The widespread use of BPS as a replacement is worrying, not only due to its similar chemical properties but also because of its resistance to biodegradation. At the same time, its relatively high polarity promotes spreading in the environment and thereby increases contamination by BPS (Frankowski et al., 2020).

BPS is being used to replace BPA in consumer goods, thermal paper, and food packaging; it is also increasingly being used in industry, including food processing, and in medicine, including dentistry (Ndaw et al., 2018; Wang et al., 2020; Togola et al., 2021). As a result, the quantity of consumer goods containing BPS is increasing sharply, and the environment, from which BPS passes into living organisms, is being contaminated. Therefore, humans are exposed to BPS through environmental contamination, inhalation, contact with products containing BPS, and ingestion of food and liquids.

The presence of BPS in human tissues and body fluids has been proven beyond a reasonable doubt (Liu et al., 2017; Smarr et al., 2018; Huang et al., 2019). Several studies have proven that BPS acts as an ED at very low concentrations (Campen et al., 2018; Nevoral et al., 2018; Řimnáčová et al., 2020; Darghouthi et al., 2022). It significantly affects hormonal control and thus disrupts reproductive processes that are completely dependent on precise hormonal regulation (summarized in Rochester and Bolden, 2015).

There is therefore an urgent need for intensive study of BPS in the field of human reproduction, specifically oogenesis and embryonic development, where there is an emerging correlation among hormonal imbalance, decreased reproductive ability and increased levels of BPS in the environment. It is necessary to focus on the study of BPS concentrations close to those present in human tissues, which are often lower than the concentrations found in the environment and are far below the concentrations that are considered toxic.

For the development of female reproductive cells *in vivo*, communication of the cumulus cells with the oocyte is essential (Fontana et al., 2020). The key phase of meiotic maturation takes place in a follicle filled with follicular fluid. Contamination of follicular fluid with EDs may result in fatal failure of oocyte development (Petro et al., 2012; Machtinger et al., 2013). The complex process of meiotic maturation may be disrupted by BPS, similar to BPA (Yang et al., 2020). Studies conducted in mouse, bovine, sheep and zebrafish models have shown, among other effects, decreased reproductive capacity (Zhang M. Y. et al., 2020), meiotic spindle disorders, altered protein expression and disrupted mitochondrial functions (Campen et al., 2018; Nevoral et al., 2018; Pan et al., 2021; Qin et al., 2021; Desmarchais et al., 2022).

In recent years, evidence proving the negative effect of BPS on human health and even on the reproductive system in both males and females has increased (summarized in Bousoumah et al., 2021; Catenza et al., 2021), although the number of studies remains limited.

The study of human oocytes and embryonic development has a number of ethical limitations, so we selected porcine oocytes as a model for our research. Their physiology is close to that of human oocytes, so it better reflects the effects of EDs on the process of meiotic maturation in human oocytes than does the most commonly used model–rodent oocytes (Eladak et al., 2015).

We determined the concentration of BPS in the follicular fluid of women (*n* = 45) and used a similar concentration as a basis for follow-up experiments. Porcine oocytes exposed to BPS exhibited defects in meiotic maturation and damage to the meiotic spindle. To the best of our knowledge, we are the first to demonstrate an increased frequency of aneuploid oocytes and impaired mitochondrial function after BPS exposure. However, the number and quality of blastocysts of oocytes that were able to complete their maturation despite exposure to BPS did not seem to be affected.

## 2 Materials and methods

### 2.1 Antibodies and chemicals

All chemicals were purchased from Sigma Aldrich (St. Louis, MO, United States) unless otherwise noted.

### 2.2 Animals and ethical statements

The authors declare that the present study was carried out in accordance with Act No 246/1992 Coll on the Protection of Animals against Cruelty under the supervision of the Animal Welfare Advisory Committee.

All the participants agreed to participate in the study and signed an informed consent after the study procedures were explained by a responsible person and all questions were answered. The patients underwent assisted reproductive technology (ART) at University Hospital and Masaryk University after approval by the Institutional Ethical Committee of University Hospital Brno (Approval No. 10-170221/EK).

### 2.3 Patient and human follicular fluid (hFF) sample collection

Forty-five patients who underwent *in vitro* fertilization/intracytoplasmic sperm injection were recruited into this study. The ages of the patients ranged from 26 to 42 years old, and the follicle number varied from 4 to 42. In this study, only patients of couples seeking infertility evaluation and treatment at the Center of Assisted Reproduction of the University Hospital were included. For each patient, basic parameters were collected, as follows: age, occupation, number of previous cycles and number of total pregnancies/pregnancies after IVF, AMH, E2 on the day of hCG, number of follicles, and number of oocytes after OPU (Supplementary Table S1). The following embryological data were collected: number of zygotes with 2 pronuclei, number of embryos, quality of embryos, type of fertilization, cleavage rate, degeneration rate after fertilization, abnormal fertilization rate (1 PN or > 2 PN), failed fertilization rate, implantation rate, biochemical positivity after ET (hCG > 5IU/L), ongoing pregnancy (gestational sac with a detectable heartbeat at 7 weeks), and live birth. Additional data, such as thermal paper exposure, stress at work and stress in personal life, residence in country/city, and live/work in risk environment (potential exposure to toxic chemicals, radiation and/or dust), were tracked.

Follicular fluid collection: Oocytes were retrieved 37 h after pregnyl administration. Only follicular fluid from the first punctured follicle was collected (to prevent contamination with blood elements or flushing medium). Follicular fluid was stored in glass tubes at −20°C until analysis.

### 2.4 Measurement of BPS levels in hFFs

Frozen samples of follicular fluid were thawed and centrifuged for 10 min (4,000 x *g*; 4°C). One milliliter of supernatant was diluted with 1 mL of 200 mM acetate buffer pH 5.4 and mixed with 20 µL of β-glucuronidase and 5 µL of 13C^12^BPS (5 ng). The mixture was incubated overnight at 37°C. The digested sample was extracted with a mixture of 2 mL acetonitrile and 3 mL ethylacetate (LC‒MS grade solvents) for 15 min at room temperature. The emulsion was centrifuged for 10 min at 2,500 x *g*, and 4 mL of the upper layer was separated and evaporated to dryness on a SpeedVac. The rest was dissolved in 75 µL of 0.1 M NaHCO_3_ and 75 µL of acetone solution with 1-methylimidazolyl-2-sulfonyl chloride was added (2 mg/mL) (Li and Franke, 2015). The sample was heated for 15 min at 60°C in a water bath, and the resulting mixture was evaporated on a SpeedVac, dissolved in 1 mL of 50% MeOH, filtered through a 0.45 µm PVDF filter and analyzed by LC‒MS.

LC‒MS/MS was performed using a NanoLC 425 UPLC system (Eksigent, Dublin, CA, United States) coupled with an AB Sciex Triple TOF 5600. Five microliters of sample was directly loaded onto a column (Synergi MAX-RP 50 μm × 0.5 mm, 4 μm × 80 Å; Phenomenex) connected to the DuoSpray ESI source at a flow rate of 20 μL/min. The total gradient time was set at 12 min. Mobile phases A (0.1% FA; 2% ACN in water) and B (0.1% FA in ACN) were used to create a separation gradient for eluting analytes with an increasing concentration of solvent B as follows: 30% for 0.5 min; 30%–95% for 6 min; 95% for 2 min; and 95%–30% for 0.5 min, followed by 3 min of equilibration at 30% solvent B. All data were acquired from the TripleTOF MS using Analyst TF 1.7 software by AB Sciex in IDA mode and processed using Multi-Quant v4 (ABSciex).

### 2.5 Oocyte collection, BPS treatment, IVM, IVF, and embryo culture

Porcine ovaries were collected from non-cycling gilts at a local slaughterhouse and rapidly transported to the laboratory at 38°C in a cooler. Cumulus-oocyte complexes (COCs) were aspirated from follicles (2–5 mm in diameter) with a 20-gauge needle fixed to a 10 mL disposable syringe. Under the stereomicroscope, only COCs that were judged to be healthy, 120 µm in diameter, with uniform ooplasm and compact cumulus mass with at least three layers of cumulus cells, were selected for further study. Suitable oocytes were cultured in modified M199 medium supplemented with sodium bicarbonate (0.039 mL of a 7.0% solution per ml of medium), calcium lactate (0.6 mg/mL), gentamicin (0.025 mg/mL), HEPES (1.5 mg/mL), 13.5 IU of the gonadotropic hormones eCG and hCG at 6.6 IU/mL (P.G.600, Intervet, Boxmeer, Netherlands) and follicular fluid (100 μL/mL) under controlled atmospheric conditions of 5% CO_2_ in air at 38°C. COCs were treated with BPS at concentrations of 3 μM, 30 nM, and 300 p.m. dissolved in dimethyl sulfoxide (DMSO) to a final concentration of 0.1%. A vehicle control of COCs cultivated in medium with the same DMSO concentration was used. The COCs were cultured for 48 h.

After IVM, cumulus cells were removed with 0.1% hyaluronidase in TL-HEPES-PVA, MII oocytes first polar body extruded were selected under a stereomicroscope, and the oocytes were washed three times with TL-HEPES-PVA and three times with mTBM (Abeydeera et al., 1998) containing 0.2% BSA. Thereafter, 15-20 oocytes were placed into each of four 100 μL drops of mTBM, which had been covered with mineral oil in a 35-mm polystyrene culture dish. The dishes were allowed to equilibrate in the incubator at 38°C. One milliliter of liquid semen preserved in BTS was washed, and spermatozoa were resuspended in mTBM medium. After the appropriate dilution, 1 μL of this sperm suspension was added to the medium containing oocytes to give a final sperm concentration of 1 × 105 spermatozoa/mL. Oocytes were coincubated with spermatozoa for 6 h at 38°C and 5% CO_2_ in air. After IVF for 5 h, oocytes were transferred into 100 μL PZM-3 medium (Yoshioka et al., 2002) containing 0.4% BSA for further culture for 144–168 h.

### 2.6 Chromosome spreads of MII pig oocytes

*In vitro* matured MII oocytes were treated with 0.5% pronase to remove the *zona pellucida*. Then, the oocytes were gently spread and fixed on SuperFrost slides as previously described (Hodges and Hunt, 2002) in a ring marked by a hydrophobic pen with fixative solution (1% paraformaldehyde; 0.15% Triton X-100; 3 mM DDT; dissolved in H_2_O) for 2–3 h in a humid chamber. The slides were used directly for immunofluorescence. After fixation, the slides were washed in PBS and blocked in 3% PBS-BSA for 60 min at room temperature (RT). Thereafter, the oocytes were incubated with CREST antiserum (Fitzgerald Industries International, 90C-CS1058; 1:800) overnight at 4°C and then washed 3 times in 1% PBS-BSA at RT before incubation with a DyLight 488-conjugated AffinityPure Donkey Anti-Human IgG (Jackson ImmunoResearch Laboratories, cat. No. 709-485-149; 1:200) for 60 min. After incubation, the slides were washed 3 times in 1% PBS-BSA and covered with cover slides with drop a of Vectashield containing 4′6-diamidino-2-phenylindole (DAPI; Thermo Fisher Scientific) for chromatin staining (Tarkowski, 1966).

### 2.7 TUNEL assay

After culturing and removing cumulus cells, oocytes and blastocysts were fixed for 30 min in 4% paraformaldehyde and treated with fluorescein-conjugated dUTP and the terminal deoxynucleotidyl transferase enzyme (*In Situ* Cell Death Detection Kit, cat. No. 11684795910, Roche, Germany) for 60 min at 38°C in the dark, in accordance with the assay protocol. A positive control was prepared in accordance with the manufacturer´s instructions. Finally, after incubation, oocytes and/or blastocysts were washed three times in PBS and mounted onto slides in Vectashield medium with DAPI. Oocytes and apoptotic cells with a positive fluorescence signal were considered TUNEL-positive cells. Images of oocytes were acquired using an Olympus IX83 spinning disc confocal microscope (Olympus, Germany). Based on DAPI observation, the fragmentation of nuclei and total nuclei in blastocysts wereobserved under a fluorescence microscope (Nikon Eclipse Ci microscope, Nikon Instruments Inc., Seoul, Korea).

### 2.8 Immunofluorescence staining and microscopy

After culture, the oocytes were fixed in a 4% paraformaldehyde solution in PBS at room temperature for 30 min. Then, the oocytes were permeabilized and blocked in 0.1% Triton X-100 (in 1% and 5% goat serum in PBS NGS-PBS) and incubated overnight with the following antibodies in 1% NGS: anti-α-tubulin (T6199; 1:1000), anti-mitofusin 1 (Abcam, ab104274; 1:200) and anti-mitofusin 2 (Abcam, ab50843; 1:200). Subsequently, the oocytes were washed 3 times in 1% NGS before incubation with fluorescein isothiocyanate (FITC)-conjugated goat anti-mouse IgG and tetramethylrhodamine isothiocyanate (TRITC)-conjugated goat anti-rabbit IgG (Thermo Fisher Scientific, Waltham, MA, United States; 1:200). Secondary antibody incubation was carried out at room temperature in the dark in 1% NGS in the dark for 40 min. After incubation, the oocytes were rinsed 3 times in 1% NGS and mounted on Vectashield containing DAPI for chromatin staining. An Olympus IX83 spinning disc confocal microscope (Olympus, Germany) was used for α-tubulin, mitofusin 1 and mitofusin 2 visualization. Image analysis was performed using ImageJ software (NIH). Signal intensities were normalized to the basal signal intensity of the negative control and compared to those in untreated oocytes. Exposure conditions were the same for each individual protein, and its negative control, which lacked a specific antibody, was processed under comparable conditions.

### 2.9 Detection of mitochondria

The mitochondrial distribution and shape of clusters were evaluated using MitoTracker Red (Thermo Fisher Scientific), which selectively stains live mitochondria. Cumulus cell-free oocytes after 48 h of IVM were incubated with 200 nM MitoTracker diluted in M199 maturation medium at 38°C for 30 min in 5% CO_2_ in air, washed 3 times and mounted in Vectashield containing DAPI. Images were acquired using an Olympus IX83 spinning disc confocal microscope (Olympus, Germany).

### 2.10 Analysis of the glycolytic activity of cumulus cells

Cumulus cells from 30 treated (bisphenol S) oocyte*-*cumulus complexes and 30 untreated (control) COCs were centrifuged for 10 min at 300 × *g* at room temperature. The pellet was resuspended in 1 mL of preheated assay medium (XF DMEM, pH 7.4, Agilent Technologies) supplemented with 1 mM sodium pyruvate, 4 mM L-glutamine and hyaluronidase at a final concentration of 0.1%. The samples were incubated for 5 min at 38°C*,* centrifuged again and resuspended in 210 µL of assay medium. The cells were transferred to an XFp cell culture miniplate coated with ≈60 μg/mL Corning^®^ Cell-Tak™ (Corning) by loading 50 µL of cell suspension into each well except the background control wells. The miniplate was then centrifuged for 5 min at 300 × *g* at room temperature to attach the cells to the coated surface. The cells were inspected under a microscope to confirm adherence. Then, another 130 µL of assay medium was added to each well, and the cells were incubated for 1 hour in a CO_2_-free incubator at 38°C. The plate was transferred to the XFp analyzer, where it recorded the changes in the extracellular acidification rate (ECAR). The glycolysis stress test was performed by introducing glucose at a final concentration of 1 g/L, stimulating maximal glycolysis with oligomycin (1 µM), and then inhibiting glycolysis with 2-deoxyglucose (18 mM). The differences in cellular responses to these additions were used to calculate different glycolytic parameters.

### 2.11 Statistical analysis

The data were obtained from at least three independent experiments and were processed with GraphPad Prism 8 (GraphPad Software Inc., San Diego, CA, United States) and Statistica Cz 12 (StatSoft, Inc., United States). Statistical comparisons were analyzed by Shapiro-Wilk normality distribution tests and analysis of variance (ANOVA). For BPA and BPS comparison, the Wilcoxon matched-pairs signed rank test was used. Tubulin abnormalities and DNA integrity analysis were performed using Fisher’s exact test with Bonferroni correction. Analysis of mitochondrial distribution and MFN1 was performed by Kruskal–Wallis followed by Dunn’s multiple comparisons test. MFN2 analysis and analysis of glycolytic activity of cumulus cells were performed using Tukey’s multiple comparison test. The remaining analyses were tested using the chi-square test. A *p*-value <0, 5 was considered statistically significant.

## 3 Results

### 3.1 BPS level in human follicular fluid (hFF)

**FIGURE 1:**
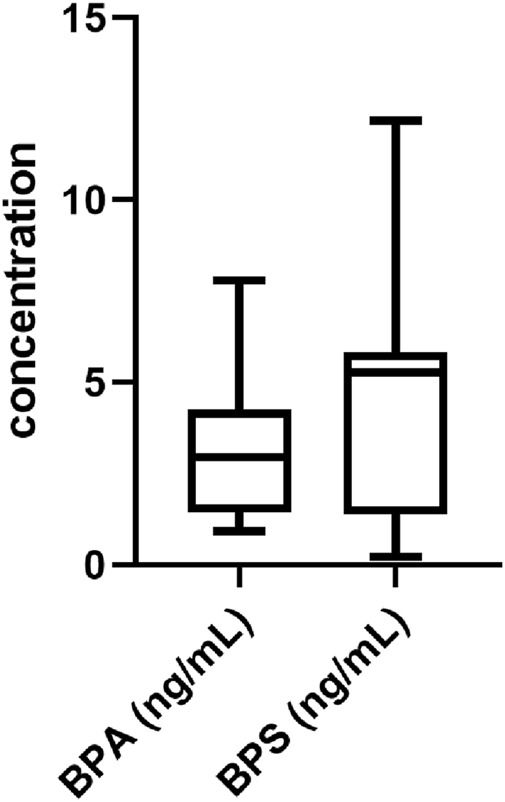
Analysis of BPS and BPA in hFF. Analysis of BPA and BPS concentrations in hFF from patients who underwent *in vitro* fertilization/intracytoplasmic sperm injection. Data are presented as box and whisker plots, where the boxes encompass values between the 25th and 75th percentiles, horizontal lines represent median values, and ‘whiskers’ give the range that includes 95% of the values. The number of samples is 45.

The goal of this experiment was to confirm the presence and evaluate the concentrations of BPS and BPA in hFF from 45 samples obtained from patients who underwent *in vitro* fertilization/intracytoplasmic sperm injection. As shown in Figure 1, BPS and BPA were present in follicular fluid. The characteristics and ART outcomes of the analyzed participants are included in the Supplementary data (Supplementary Table S1). The concentrations of BPA and BPS were measured in the ranges of 0.92–7.80 ng/mL and 0.69–12.16 ng/mL, respectively.

The average follicular content of BPS was 5.13 ng/mL (equivalent to 22.4 nM), and from this average value, the total concentrations of BPS (300 p.m., 30 nM or 3 µM) used in subsequent experiments were derived.

### 3.2 The Effect of BPS exposure on the progression of meiotic maturation and developmental competence of porcine oocytes

**FIGURE 2:**
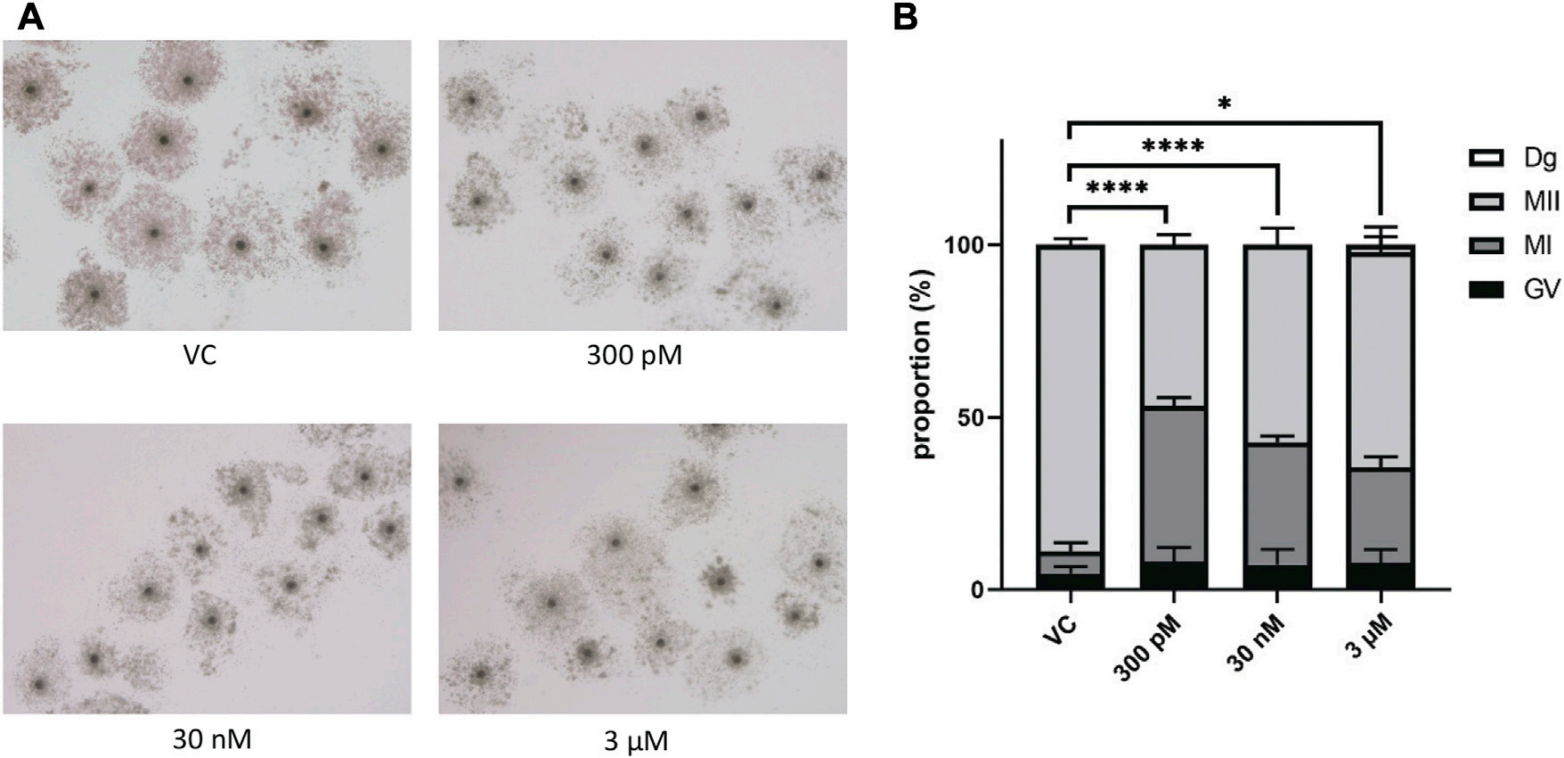
Effects of BPS on the meiotic maturation of porcine oocytes. **(A)** Representative images of cumulus expansion after 48 h of *in vitro* treatment with BPS (0, 300 p.m., 30 nM, or 3 µM). Bar, 500 μm; 3 µM. **(B)** Oocyte maturation rates of the control and BPS-treated groups (300 p.m., 30 nM, and 3 μM) based on the stages of meiotic maturation achieved by oocytes after 48 h *in vitro*: GV, germinal vesicle, MI, metaphase I, and MII, metaphase II. * Significantly different (*p* < 0.05); **** (*p* < 0.0001). The data are expressed as the mean ± SEM from four independent experiments, *n* = 100 oocytes.

We first evaluated the effect of BPS on porcine oocyte maturation to metaphase II (MII) and polar body extrusion. Cumulus–oocyte complexes (COCs) were treated with different concentrations of BPS (300 p.m., 30 nM or 3 µM) for 48 h (Figure 2A). As shown in Figure 2B, all concentrations used significantly decreased the proportion of cells that reached the MII stage. Some of the oocytes did not initiate meiotic maturation and remained in the germinal vesicle (GV) stage (4.7% ± 2.05% for the control vs. 8.0% ± 4.27% for 300 p.m. BPS; 5.3% ± 3.05% for 30 nM BPS; and 4.3% ± 1.87% for 3 µM BPS) or were assessed as degenerated (2.3% ± 3.1 for 3 µM BPS). Some of the oocytes in the exposed groups were able to initiate meiotic maturation but remained arrested in the metaphase I (MI) stage (6.3% ± 2.58% for the control vs. 45.3% ± 2.32% for 300 p.m. BPS; 36.3% ± 2.14% for 30 nM BPS; and 21.6% ± 1.63% for 3 µM BPS). Most of the oocytes finished meiotic maturation and achieved the MII stage (89% ± 1.69% for the control vs. 46.7% ± 2.79% for 300 p.m. BPS; 58.4% ± 3.80% for 30 nM BPS and 71.7% ± 0.91% for 3 µM BPS). All these differences were significant (*p* < 0.05 and *p* < 0.0001, *n* = 100 oocytes in each group). These data demonstrated a significant dose-dependent decrease in MII stage achievement after 48 h of *in vitro* culture.

Subsequently, we focused on the development of oocytes that successfully completed meiotic maturation and extruded the first polar body. No significant influence on the number of blastocysts was detected (Supplementary Figure S2A), and the quality of blastocysts or the number of double strand breaks (DSBs) in blastocysts was detected (Supplementary Figures S2A, S2B).

### 3.3 BPS exposure led to spindle defects and chromosome misalignment in porcine oocytes

**FIGURE 3:**
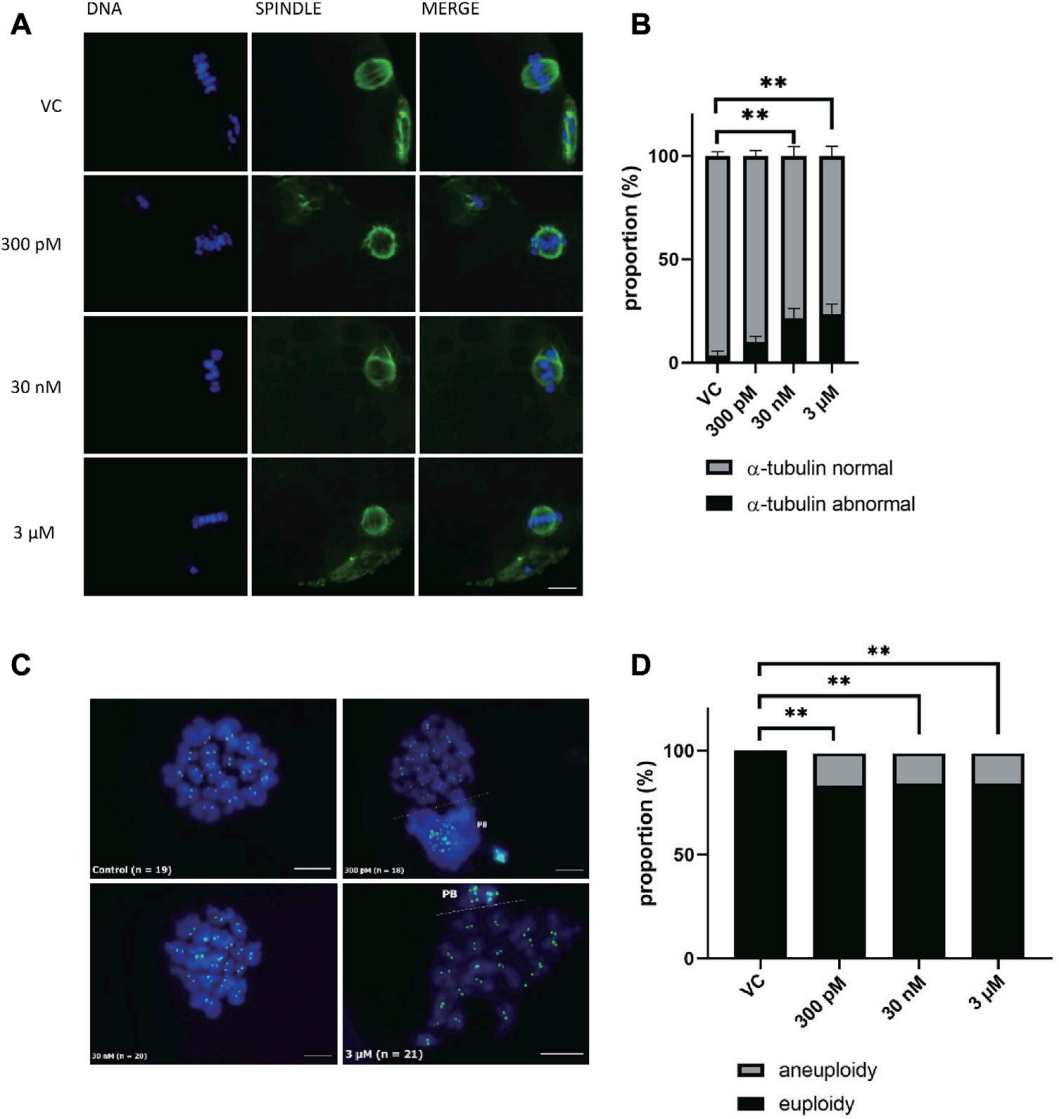
Effect of BPS meiotic spindle and chromosome alignment. **(A)** Representative images of spindle morphology and chromosome alignment after BPS treatment (0, 300 p.m., 30 nM, or 3 µM) for 48 h α-tubulin, green; DNA, blue; Bar, 10 µm. **(B)** The rate of defects in the morphology of spindle organization was quantified for the control and BPS groups. ** Significantly different (*p* < 0.01); *** (*p* < 0.001). The data are expressed as the mean ± SEM from three independent experiments, *n* = 80 oocytes. **(C)** Representative images of chromosomes in control or BPS-treated oocytes matured *in vitro* (300 p.m., 30 nM, or 3 µM). PB–dispersed polar body chromosomes; CREST, green; DNA, blue; Bar, 10 µm. **(D)** Quantification of the aneuploidy rate after 48 h of *in vitro* culture with or without BPS. **** Significantly different (*p* < 0.05). The data are expressed as the mean ± SEM from eight independent experiments, *n* = at least 23 oocytes.

Oocyte maturation requires the correct assembly of cytoskeletal structures. Considering that BPS disrupts meiotic maturation, we next examined spindle organization and chromosome alignment in oocytes after 48 h of culture. We investigated whether BPS disrupted the normal structure of the meiotic spindle and increased the percentage of oocytes with an abnormal tubulin configuration compared with the control group of oocytes with a typical barrel-shaped spindle morphology and well-aligned chromosomes in the metaphase plate. A significantly increased frequency of spindle disorganization and chromosome misalignment was observed in all BPS groups, and this effect was dose dependent (10% ± 0.58% for 300 p.m. BPS; 21.3% ± 0.61% for 30 nM BPS and 23.8% ± 0.41% for 3 µM BPS vs. 3.7% ± 0.58% for the control). The differences observed for 30 nM and 3 µM BPS were statistically significant (*p* < 0.01 and *p* < 0.001, respectively, *n* = 80 oocytes in each group) (Figure 3B). As shown in the representative images in Figure 3A, abnormal chromosome alignment (i.e., irregular or misaligned chromosomes, formation of clusters and doubled metaphase figure), as well as defects in spindle organization (multipolar spindle, disperse spindle, asymmetric and disturbed tubulin filaments), were observed. All these defects were defined as abnormal morphology and indicated poor oocyte quality.

### 3.4 BPS exposure caused oocyte aneuploidy

BPS exposure led to chromosome misalignment and defects in microtubule spindle organization, which are very important in chromosome segregation. Therefore, we postulated that numerical abnormalities of chromosomes occurred during meiotic maturation in oocytes after BPS exposure. Thus, our next step was to analyze the karyotype of MII oocytes by chromosome spreads and kinetochore labeling. Representative pictures of karyotypes in the control and BPS-exposed groups are shown in Figure 3C. As shown in Figure 3D, all concentrations of BPS increased the number of aneuploidy oocytes. In contrast to the control group (without any aneuploid oocytes), the BPS-treated oocytes showed a significantly elevated rate of aneuploidy, which was similar in all experimental groups treated with BPS 21.7% ± 0.44% for 300 p.m., *n* = 27; 18.6% ± 0.33% for 30 nM, *n* = 32, and 22.9% ± 0.41% for 3 μM, *n* = 23, *p* < 0.05.

### 3.5 BPS exposure did not affect the DNA integrity of porcine oocytes

**FIGURE 4:**
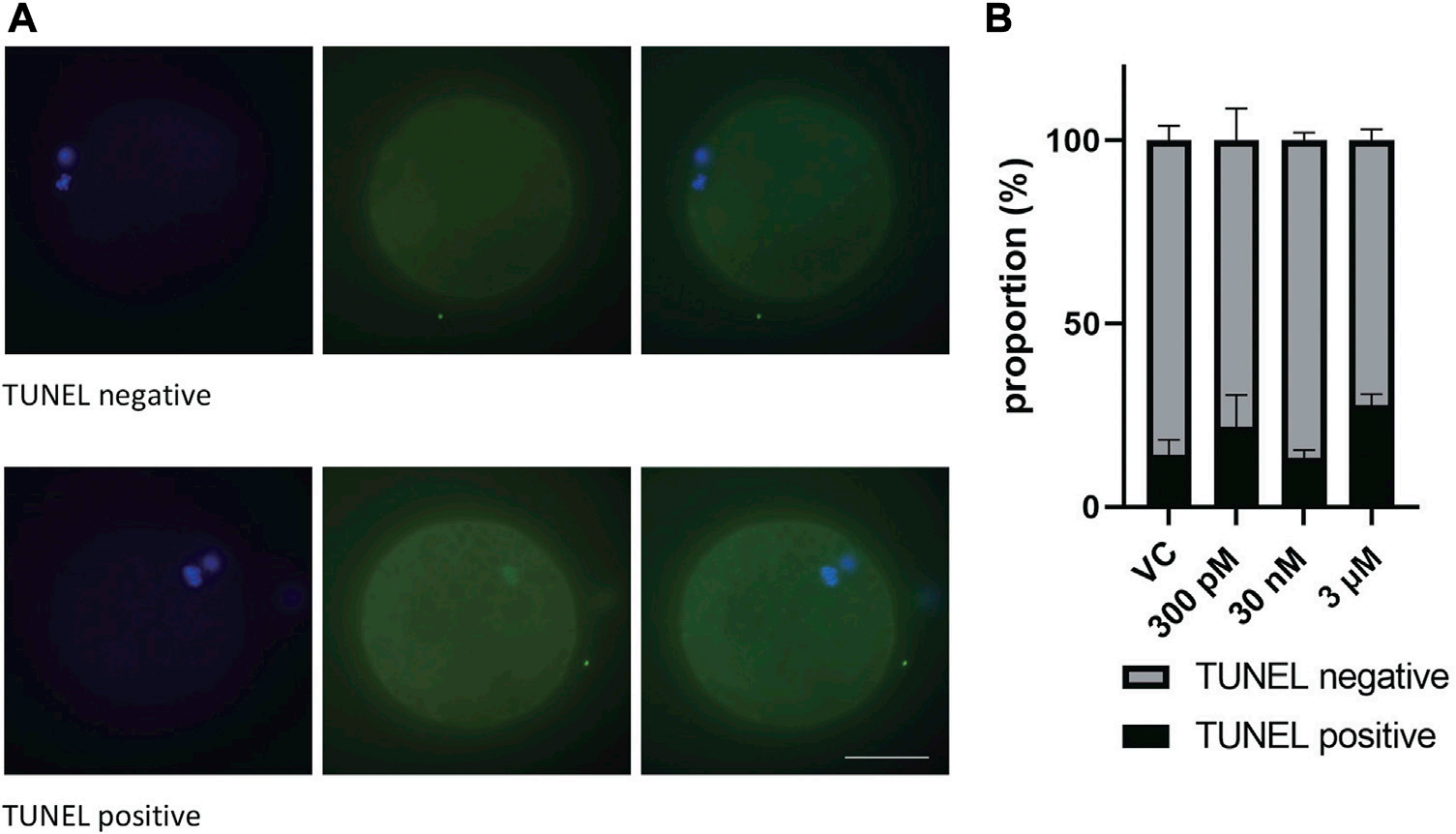
The effect of BPS on the DNA integrity of mature porcine oocytes by analysis of DNA DSBs. **(A)** Representative images of TUNEL-positive and TUNEL-negative oocytes. DSBs green; DNA, blue. Bar, 50 µm **(B)** Proportion of TUNEL-positive/negative oocytes in the control and BPS-treated groups (300 p.m., 30 nM, or 3 µM). The data are expressed as the mean ± SEM from three independent experiments, *n* = at least 50 oocytes. *p* < 0.05 (*).

Considering that BPS alters the number of chromosomes in oocytes, we decided to detect DNA fragmentation in these oocytes by TUNEL assays to analyze DNA double-strand breaks (Figure 4A). Surprisingly, none of the differences were significant. It seems that BPS does not cause DNA damage or cell death, as shown in Figure 4B. Similarly, no significant influence on the quality of blastocysts or the number of double strand breaks (DSBs) in blastocysts was detected (Supplementary Figures S2A, S2B).

### 3.6 BPS exposure changed the pattern of mitochondrial distribution in MII-Stage oocytes

**FIGURE 5:**
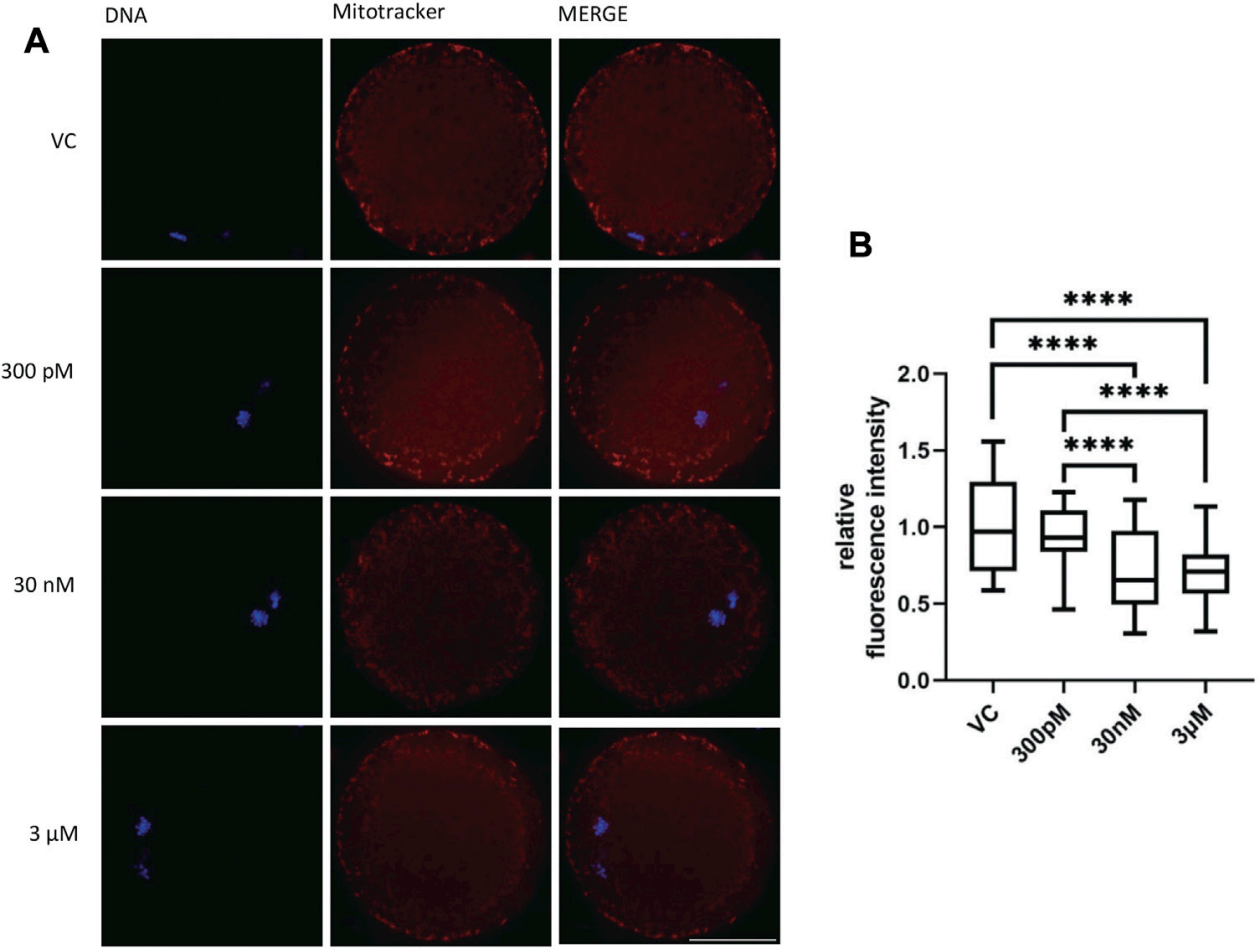
The effect of BPS on mitochondria in porcine oocytes after 48 h of exposure to BPS. **(A)** Representative images of the mitochondrial distribution in oocytes. MitoTracker, red; DNA, blue; Bar, 50 µm. **(B)** Quantification of the fluorescence intensity of mitochondria in oocytes. The data are expressed as the mean ± SEM from four independent experiments, *n* = at least 60 oocytes. Significantly different *p* < 0.0001 (****).

Mitochondria are indispensable for meiotic maturation and play an important role in the regulation of cell death. Consequently, the next step was to evaluate the influence of BPS on mitochondrial distribution and numbers in BPS-exposed oocytes. We noticed changes in the mitochondrial pattern in some oocytes after 48 h of BPS treatment. Whereas in the control group, the distribution of mitochondria was mainly homogenous in the cytoplasm and cortical area, in BPS-exposed oocytes, some irregularities in localization appeared (accumulation in a specific part of the ooplasm and reduced number of mitochondria in the peripheral area of oocytes) (Figure 5A). The fluorescence intensity of the oocyte images was measured and expressed relative to that of the control group (1 ± 0.03, *n* = 86). In comparison to that of the control oocytes, the relative intensity of mitochondria in BPS-treated oocytes was lower. The differences were statistically significant at higher doses of BPS (0.73 ± 0.03 for 30 nM, *n* = 70 and 0.70 ± 0.02 for 3 μM, *n* = 72). The decrease in the relative intensity of mitochondria was not significant in oocytes exposed to 300 p.m. BPS (0.91 ± 0.02, *n* = 60), *p* < 0.0001 (Figure 5B).

### 3.7 BPS exposure causes changes in mitofusin patterns in porcine oocytes

**FIGURE 6:**
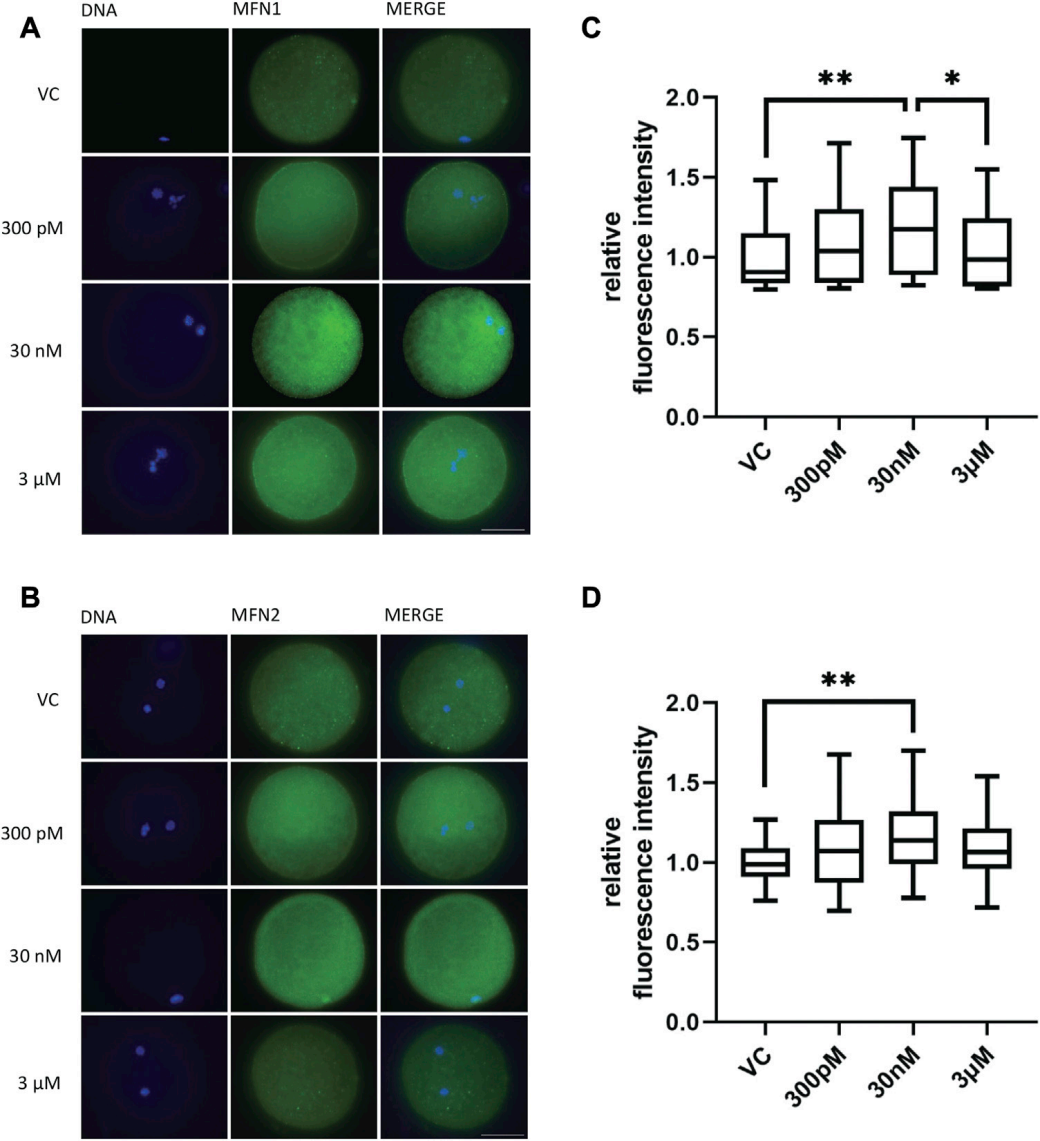
The effect of BPS on the mitochondrial fusion proteins MFN1 and MFN2 in porcine oocytes after 48 h of *in vitro* maturation. **(A)** Representative images of MFN 1 distribution in oocytes. MFN1, green; DNA, blue. Bar, 50 µm. **(B)** Representative images of MFN2 distribution in oocytes. MFN2, green; DNA, blue. Bar, 50 µm **(C)** Quantification of the fluorescence intensity of MFN1 in the control and BPS-exposed groups. The data are expressed as the mean ± SEM from three independent experiments, *n* = at least 60 oocytes; asterisks denote the statistical significance of differences among experimental groups (*p* < 0.05). **(D)** Quantification of the fluorescence intensity of MFN2 in the control and BPS-exposed groups. The data are expressed as the mean ± SEM from three independent experiments, *n* = at least 40 oocytes; asterisks denote the statistical significance of differences among experimental groups at *p* < 0.05 (*) and 0.01 (**).

After determining the effect of BPS exposure on the number of mitochondria, we were interested in the influence of BPS on mitochondrial fusion proteins (mitofusins), which play an important role in meiotic maturation. The aim was to evaluate the influence of BPS on the quantity and distribution of mitofusin 1 (MFN1) and mitofusin 2 (MFN2) in oocytes after 48 h of *in vitro* culture. The relative fluorescence intensity increased for MFN 1 in the 30 nM BPS-exposed group (1.19 ± 0.04 for 30 nM *n* = 53 vs. 1 ± 0.03 for the control, *n* = 42, *p* < 0.01; 1.19 ± 0.04 for 30 nM BPS vs. 1.04 ± 0.03 for 3 µM BPS, *n* = 46, *p* < 0.05). MFN2 expression was significantly increased in the group exposed to 30 nM BPS (1.17 ± 0.04, *n* = 45 vs. 1 ± 0.02 for the control, *n* = 36, *p* < 0.01) (Figures 6C, D). As shown in Figures 6A, B, the intracellular localization of both mitofusins was also changed. We noticed accumulation of mitofusins in clusters in the cytoplasm of oocytes.

### 3.8 Effect of BPS on the glycolytic activity of cumulus cells

The oocyte and its surrounding cumulus cells regulate each other’s metabolic function to support oocyte maturation. Glucose is the driver of cumulus cell metabolism and is essential for ATP production, whereas mitochondria are the primary source of ATP. Because of the described changes in the mitochondria of oocytes, we also analyzed the glycolytic activity of cumulus cells surrounding the oocyte during meiotic maturation *in vitro*. A significant difference in the groups exposed to BPS compared to the control was not observed (Supplementary Graphs S1A–S1D).

## 4 Discussion

The small number of existing studies related to BPS effects in very low doses was based on BPS concentrations measured mostly in human urine (up to 6.7 ng/mL), serum (0.15 ng/mL), or seminal plasma (up to 0.85 ng/mL) (Thayer et al., 2016; Jin et al., 2018; Smarr et al., 2018; Ješeta et al., 2022). The only study that confirmed the presence of BPS in follicular fluid (FF) was aimed at investigating its effect on somatic cells (Amar et al., 2020). Therefore, we decided to focus on the effect of BPS on the development of oocytes at concentrations that correspond to the *in vivo* dose of BPS present in the hFF of patients undergoing assisted reproduction.

We confirmed that BPS is present in the hFF of patients who have undergone IVF treatment. The presence of BPS in the hFF is likely to affect oocyte development *via* a mechanism similar to that described for BPA (summarized in Rochester and Bolden, 2015; Poormoosavi et al., 2019). As demonstrated by Amar et al. (2020), BPS affects steroidogenesis in human granulosa cells by disrupting the secretion of progesterone and estradiol hormones. The level of BPS detected by us, 22.4 nM, is higher than the level of BPA that is currently regulated or banned. The presence of BPS in hFF could impair human oogenesis. However, direct verification of this hypothesis in human oocytes is restricted by a number of ethical issues. Nevertheless, the porcine oocyte model is generally considered adequate for assessing such situations (Mordhorst and Prather, 2017). Our results may reflect the effect of BPS on human oocytes better than studies conducted on rodent species due to the higher sensitivity of humans to the effects of bisphenols[rodent oocytes are at least 100-fold less sensitive to BPA than human oocytes–Eladak et al. (2015)].

Based on these facts, we selected BPS concentrations of 300 p.m., 30 nM (corresponding to the dose detected in hFF), and 3 μM and evaluated their effect on the success of meiotic maturation in porcine oocytes. The ability to reach the MII stage and to extrude the polar body was significantly reduced in all experimental groups, which is consistent with our previous research performed on BPS (Žalmanová et al., 2017). We assume that the failure of meiotic maturation is caused by the disruption of microtubule dynamics (Mullen et al., 2019). This hypothesis was confirmed by observations of poorly anchored tubulin fibers, acentric positioning of the meiotic spindle poles and acentric arrangement of chromosomes, as well as observations of chromosomes outside the metaphase figure and an increased or reduced amount of chromatin. This conclusion is supported by studies performed with other bisphenol variants–BPA, BPAF, BPB, and BPF (Machtinger et al., 2013; Ding et al., 2017; Campen et al., 2018; Zhang S. X. et al., 2020; Nevoral et al., 2021).

In the case of BPA, an association with aneuploidy, which is a frequent cause of human infertility, has been demonstrated (Hunt et al., 2003; Susiarjo et al., 2007; Eichenlaub-Ritter et al., 2008). In the study of George et al. (2008), abnormal tubulin arrangement was associated with aneuploidy in the presence of BPA. This suggested that BPS might also be involved in the induction of aneuploidy. To the best of our knowledge, our study is the first to prove a link between BPS exposure and the formation of aneuploid mammalian oocytes. Aneuploidy was detected at all tested levels of BPS, including the concentration of BPS measured by us in the hFF of IVF patients. The aneuploidy incidence in the human population is therefore increased not only by the higher age of reproductively active females (Nagaoka et al., 2012; summarized in Ma et al., 2020) but also by increasing exposure to endocrine disruptors, such as BPS.

We also asked whether apoptosis induced by BPS, which has been observed in bovine (Saleh et al., 2021) and mouse (Prokešová et al., 2020) oocytes, could be the mechanism underlying aneuploidy. In porcine oocytes exposed to BPS, however, the increased disruption of chromatin integrity, which could ultimately lead to oocyte apoptosis, was not statistically significant.

We non-etheless decided to examine the possible association of BPS with cellular stress by monitoring the expression of mitochondrial proteins, with which BPA is known to be able to interfere (Pan et al., 2021). Porcine oocytes exposed to BPS exhibited different patterns of mitochondria at concentrations of 30 nM and 3 μM. To closely describe the effect of BPS in oocytes, we selected the MFN1 and MFN2 proteins for analysis. The key role of these mitochondrial fusion proteins in oocytes was discovered only recently. In mice, MFN1 is involved in oocyte growth and communication with follicular cells (Carvalho et al., 2020). Deletion of the MFN2 gene in mouse oocytes leads to mitochondrial dysfunction and female subfertility associated with impaired oocyte maturation and follicular development (Zhang et al., 2019). Based on our analysis, porcine oocytes exposed to BPS exhibit changes in the expression of both of these proteins. Statistically significant changes in MFN1 expression were observed at a dose of 30 nM BPS in comparison to the control and 3 μM BPS-exposed groups, and changes in MFN2 expression were observed at a dose of 30 nM BPS compared to the control group. Mitofusin function impairment by the presence of BPS in the follicular fluid can lead to defects in meiotic maturation, which may be causes of the declining reproductive capacity of both humans and livestock.

Mitochondrial dysfunction affects energy production in oocytes but not in cumulus cells, which are thought to rely mainly on the glycolysis for energy (Kansaku et al., 2017). Therefore, we tested the impact of BPS on glycolytic activity of cumulus cells. However, no significant effect on the glycolytic activity of cumulus cells has been confirmed.

We also focused on IVF and early embryonic development. In the oocytes that were able to extrude the PB and reach stage II of meiotic division, there was no difference in the number or quality of blastocysts.

## 5 Conclusion

The results of our study demonstrate that the exposure of oocytes to BPS at doses corresponding to measured contamination in hFF interferes with the meiotic maturation and quality of porcine oocytes. To the best of our knowledge, this is the first study suggesting the impact of BPS on the occurrence of aneuploidy and impaired function and dynamics of mitochondria in oocytes. Moreover, our results suggest that BPS is not a safer alternative to BPA and that its increasing production is an underestimated threat to human reproduction.

## Data Availability

The raw data supporting the conclusion of this article will be made available by the authors, without undue reservation.
