# Supplementary material for: The bisphenol S contamination level observed in human follicular fluid affects the development of porcine oocytes
Source: Front Cell Dev Biol. 2023 Apr 6;11:1145182. doi: 10.3389/fcell.2023.1145182 (PMC10115966; doi:10.3389/fcell.2023.1145182)
Supplement: Supplementary file 1 [file DataSheet1.PDF]

Table S1: Participant characteristics and ART outcomes.

characteristics	average	Median	SD	Min-max
Age (years)	33.1	33.5	4.5	26-42
Number of previous cycle	0.6	0	0.9	0-3
E2 (pg/ml)[a]	6.0	5.4	3.6	0.6-16.1
AMH (pg/ml)[b]	4.0	3.4	3.1	0.4-11.9
Number of follicles	15.9	15.0	7.1	4-42
Number of oocytes	9.1	9.0	4.1	2-17
Number of zygotes	6.7	7.0	3.8	0-14
Number of abnormal embryos[c]	2.1	2.0	2.1	0-10
Number of usable embryos[d]	4.6	4	2.6	0-9
Biochemical pregnancies	11			
Clinical pregnancies	9			
Live birth	2			

[a]serum estradiol concentration at the day of OPU

[b]serum anti-Müllerian hormone concentration at the day of OPU

[c]degeneration rate after fertilization, abnormal fertilization rate (1PN or >2PN)

[d]blastocysts capable to be transfered

Graph S1: Changes in glycolytic parameters after exposure to BPS.

A

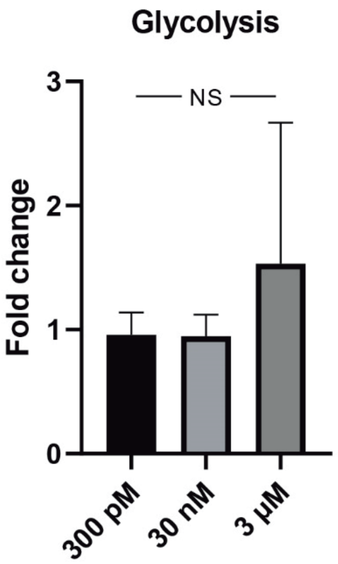

B

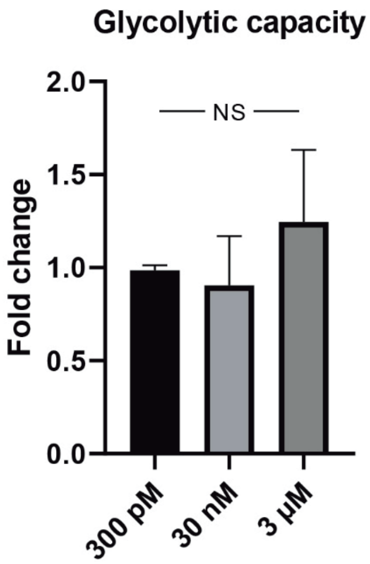

C

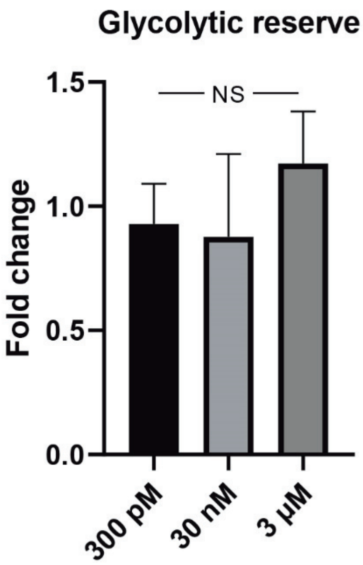

D

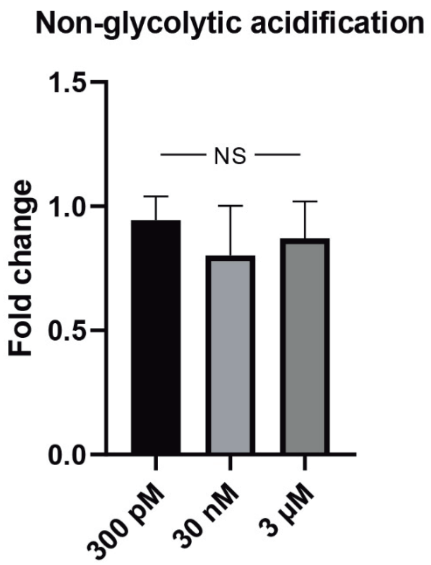

A) Glycolytic rate is measured as extracellular acidification minus non-glycolytic acidification.

B) Glycolytic capacity is maximum glycolytic rate

C) Glycolytic reserve is the difference between maximum glycolytic rate and basal glycolysis.

D) Non-glycolytic acidification

All rates are expressed as fold change from control ± SD.

Table S2: Effect BPS on blastocyst achievement and developmental quality of blastocyst.

Table S2A. BPS effect on blastocyst achievement and the quality after exposure of mature oocyte

	Vehicle control	300 pM	30 nM	30 µM
Cleavage (%)[a]	58.1 ± 5.6	61.1 ± 9.0	58.3 ± 5.7	56.7 ± 4.2
Blastocyst achievement (%)[b]	8.9 ± 6.0	12.9 ± 6.2	8.9 ± 3.8	9.8 ± 4.3
Cell 3No. per blastocyst	23.75 ± 2.10	28.20 ± 2.62	35.00 ± 7.77	25.75 ± 2.02
Fertilized oocytes (n)	61	65	57	59

[a]Cleaved embryo per fertilized oocytes

[b]Proportion of blastocyst per cleaved embryos

Table S2B. BPS effect on apoptosis and fragmentation of blastocyst after exposure of mature oocyte.

	Vehicle control	300 pM	30 nM	30 µM
No. apoptotic cell per blastocyst (%)	3.50 ± 0.96 (14.2 ± 2.8)	1.40 ± 0.75 (5.8 ± 3.4)	2.33 ± 0.88 (6.3 ± 1.2)	0.75 ± 0.25 (2.9 ± 1.0)
No. cell fragmentation per blastocyst (%)	3.25 ± 1.25 (12.8 ± 3.5)	2.20 ± 1.11 (8.7 ± 5.0)	1.67 ± 0.33 (4.8 ± 0.2)	2.75 ± 1.11 (11.8 ± 5.0)
Blastocyst (n)	4	5	3	4

Mature oocytes were treated with BPS. Developmental competence was evaluated using the recording of cleavage and blastocyst rate after 24 and 96 hrs, respectively, following the *in vitro* fertilization with untreated sperm and further embryo culture. Moreover, apoptotic and fragmented blastomeres in blastocysts were counted as qualitative parameters of embryos. There is neither observable effect of used doses of BPS on the competence of the first mitotic cleavage nor blastocyst achievement (Table S2A). Similarly, the quality of blastocyst was not affected and the rate of apoptotic blastomeres did not exceed the physiological threshold of vehicle control (Table S2B).
